# Inattentional blindness in medicine

**DOI:** 10.1186/s41235-024-00537-x

**Published:** 2024-03-27

**Authors:** Connor M. Hults, Yifan Ding, Geneva G. Xie, Rishi Raja, William Johnson, Alexis Lee, Daniel J. Simons

**Affiliations:** University of Illinois at Champaign-Urbana, Champaign, USA

## Abstract

People often fail to notice unexpected stimuli when their attention is directed elsewhere. Most studies of this “inattentional blindness” have been conducted using laboratory tasks with little connection to real-world performance. Medical case reports document examples of missed findings in radiographs and CT images, unintentionally retained guidewires following surgery, and additional conditions being overlooked after making initial diagnoses. These cases suggest that inattentional blindness might contribute to medical errors, but relatively few studies have directly examined inattentional blindness in realistic medical contexts. We review the existing literature, much of which focuses on the use of augmented reality aids or inspection of medical images. Although these studies suggest a role for inattentional blindness in errors, most of the studies do not provide clear evidence that these errors result from inattentional blindness as opposed to other mechanisms. We discuss the design, analysis, and reporting practices that can make the contributions of inattentional blindness unclear, and we describe guidelines for future research in medicine and similar contexts that could provide clearer evidence for the role of inattentional blindness.

## Introduction

Failures of awareness when providing patient care can have devastating consequences, but case studies suggest they might be distressingly common. In one case, for example, a 15 cm epidural catheter fragment was left in a patient following a procedure, and it went unnoticed for 12 years despite multiple opportunities to detect it in X-rays and CT scans (Pinciroli & Fumagalli, 2015). Similarly, radiologists, emergency physicians, and intensivists failed to spot a retained guidewire in chest radiographs and CT scans (Lum et al., 2005). One nurse missed signs of a heart attack despite detailed documentation of the patient’s dropping blood pressure in their medical records (Jones & Johnstone, 2017). And physicians missed evidence of air within the skull in one case and a collapsed lung in another when they were using CT scans to confirm the placement of hardware prior to surgery (Park et al., 2021).

Doctors or technicians looking at a scan or patient for one problem often miss other issues that were obvious in hindsight. For example, a person who had been diagnosed with tuberculosis died from undiagnosed lymphoma, possibly because the cancerous cells looked similar to a typical presentation of tuberculosis in a lymph node biopsy (Owattanapanich et al., 2017). Similarly, 11 out of 12 ophthalmologists missed signs of iron toxicity from a metallic object when asked to look for and rule out malignant melanoma (Zamir, 2015), and only about 25% of radiological reports mentioned the presence of an additional rib during their initial read of a CT scan taken for other purposes (Viertel et al., 2012). In an analysis of initial readings of radiological images that resulted in delayed diagnosis, approximately 42% of the errors were attributed to “underreading” or missing the finding, and 82% of the examined cases had an instance of underreading (Kim & Mansfield, 2014). A prospective study using MRI scans of 60 patients with known lesions (Garg et al., 2022) showed that neuroradiologists performing a routine read missed the presence of lesions in 21 patients (including 6 patients with multiple lesions). On average, the two radiologists who were instructed to look for that particular form of lesion found at least one in 55.5 out of 60 patients.

A common thread in such case studies is the possible role of inattentional blindness: the failure to notice fully visible but unexpected objects when focusing attention on a task (Mack & Rock, 1998). For example, when people watch a video and count how many times a group of people wearing white shirts pass a ball (while ignoring passes by people wearing black shirts), about half of them fail to notice a person in a gorilla suit unexpectedly walk through the scene (Simons & Chabris, 1999; see also Neisser, 1979).

Relatively few inattentional blindness studies have examined noticing in complex, real-world contexts like medical diagnosis or radiology. Instead, most studies adopt one of two laboratory approaches. In a typical *transient* inattentional blindness task, people miss a briefly flashed unexpected object while they perform a rapid judgment about another object. For example, in studies by Mack and Rock (1998), who coined the term inattentional blindness, people first complete several trials in which they judge which arm of a briefly flashed cross is longer: the horizontal or vertical. Then on a critical trial, in addition to the cross, another object unexpectedly appears. Immediately after the trial, participants are asked if they noticed anything other than the cross, and under typical conditions, anywhere from 25 to 75% of participants miss it.

In *sustained* inattentional blindness tasks (like the “gorilla” video), people pay attention to a subset of moving objects, and many fail to notice an additional moving object. Most sustained inattentional blindness studies now adopt better-controlled, computerized variants of the basketball pass-counting task (e.g., Most et al., 2000). For example, participants might track black shapes or letters and ignore white ones as they bounce around on a computer screen for 10–20 s. After each trial, participants report the number of times the attended objects bounced off the edges of the screen. On the critical trial, an unexpected object enters on one side of the screen, moves across the display, and exits the other side of the screen. And as in the transient task, participants are asked if they noticed it.

In both transient and sustained tasks, the critical trial is sometimes followed by a divided attention trial, where participants know that something “unexpected” might appear while they still perform the primary task. And, some studies include a final, full-attention trial designed to ensure that participants can detect the object if their only task is to look for it (Mack & Rock, 1998).

Salient, unique objects often go unnoticed in both transient and sustained tasks. For example, when attending to white or black objects in a sustained inattentional blindness task, about 30% of participants missed an unexpected red cross (Most et al., 2001). Unexpected objects that are more similar to the attended items and more distinct from the ignored ones tend to be noticed more frequently (e.g., Ding et al., 2023; Goldstein & Beck, 2016; Most et al., 2001, 2005; Simons & Chabris, 1999; Wood & Simons, 2017). For instance, when attending to black objects and ignoring white ones, an unexpected dark gray object, closer in luminance to the attended black objects, was noticed more than an unexpected light gray object, which was more similar to the ignored white objects (Most et al., 2001). The pattern reversed when attending to white and ignoring black (unexpected light gray objects were noticed more). Hence, instances of inattentional blindness seem to be influenced not only by the physical characteristics of the unexpected object, but also by top-down filtering of task-relevant from irrelevant information.

Although most studies of inattentional blindness adopt computer- or video-based tasks, several have examined noticing under more natural, real-world conditions. In one study (Chabris et al., 2011), participants who jogged behind an experimenter and counted how many times the experimenter touched their head often failed to notice a fistfight staged along their route. Many pedestrians missed a clown unicycling near their path or money attached to a tree, especially if they were talking on a mobile phone at the time (Hyman et al., 2010; Hyman Jr. et al., 2014) People can even miss real objects that have practical consequences: Approximately 58% of police trainees and 33.3% of experienced police officers failed to notice a gun visible on the dashboard of a car during a simulated traffic stop (Simons & Schlosser, 2017).

Medical case studies of failures of awareness suggest that inattentional blindness might contribute to missed diagnoses and problems that have direct, practical consequences for patients. The same principles that apply to laboratory and real-world studies of inattentional blindness likely apply in medical contexts as well. Objects like a retained guidewire are obvious when radiologists search for them, but easily missed when focusing attention elsewhere. And, if radiologists are searching for something that would appear light on a radiograph, they might be more likely to notice unexpected light-colored anomalies than dark ones.

Not all failures of awareness are instances of inattentional blindness; there are many other reasons why doctors, nurses, or other medical professionals might fail to notice an incidental finding. Inattentional blindness refers specifically to a failure to notice a fully visible, unexpected object when people are focusing their attention on something else. Only if an event is unexpected can we be sure that participants were not deliberately devoting some attention to it (Mack & Rock, 1998). If people know that an object might appear, they will deliberately devote some of their limited attention to detecting it, making it a divided attention task rather than a test of inattentional blindness. In many cases, the design of a study can make it difficult to determine whether or not the critical object was truly unexpected. In terms of the practical consequences for a patient, whether or not a problem was missed due to inattentional blindness or due to a different sort of awareness failure is immaterial. But the distinction might well be relevant in setting policy or changing practices to help reduce errors.

Inattentional blindness methods were devised to examine how much processing can or cannot occur in the complete absence of attention (Mack & Rock, 1998). Studies dating back to the original dichotic listening work in the 1950s (e.g., Cherry, 1953) demonstrated how easily we can miss one event because we are paying attention to something else. And techniques like dichotic listening were used to argue that the ignored elements are unattended (e.g., Corteen & Wood, 1972; examples of the cocktail party effect: Cherry, 1953; Moray, 1959). However, such methods cannot fully rule out the possibility that people are devoting some attention to the “ignored” information and processing it with some degree of attention (Holender, 1986). Inattentional blindness tasks overcome that objection by ensuring that the critical object is unrelated to the primary task people are performing so that they have no reason to devote attention to it (even to ignore it); the critical object must be unexpected so that participants have no reason to devote attention to its possible appearance. This approach means inattentional blindness studies typically can only use a single critical trial or event. Once people know that something additional might appear, they will devote some attention to that possibility, making the task one of divided attention.

The boundary between failures of awareness under conditions of divided attention and those due to inattentional blindness might seem trivial, but it might also reflect entirely distinct mechanisms. Other evidence suggests that the ability to ignore or filter irrelevant information might rely on separate mechanisms from those affecting noticing of unexpected objects. In fact, noticing under conditions of inattentional blindness might not be an *ability* at all. Measures of cognitive ability reliably predict individual differences in the sorts of attentional control mechanisms involved in divided or selective attention task performance, but those same measures do not seem to predict noticing of unexpected objects in inattentional blindness tasks (Simons et al., 2024). For example, measures of attentional control and working memory such as OSPAN predict performance on a wide range of attentional control tasks, including the attentional blink (Willems & Martens, 2016), negative priming (Conway et al., 1999), and attention capture (Unsworth et al., 2004).

In a medical context, a radiologist looking to determine whether or not a radiograph includes a lesion might stop their search once they find one, leading them to miss an unrelated problem (or even another lesion) in the same image (e.g., Berbaum et al., 2013; for a recent review and discussion, see Adamo et al., 2021). That tendency to miss a second target is associated with individual differences in performance on measures of vigilance and other measures of attentional performance (Adamo et al., 2017). Similarly, individual differences in conscientiousness predicted misses on a visual search task for both experienced and early-career airport baggage scanners (Biggs et al., 2017). But a meta-analysis of 38 articles with a total of 74 distinct individual difference samples found little evidence of an association between standard measures of cognitive ability and noticing of unexpected objects in inattentional blindness tasks (Simons et al., 2024). None of the cognitive ability measures that typically are associated with better performance on selective attention tasks (e.g., span tasks, measures of fluid intelligence, flanker tasks) strongly predicted who would or would not notice unexpected objects.

That distinction has practical consequences. For situations in which individual differences in performance on cognitive tasks or personality predict noticing of critical objects, we potentially could select for those traits or abilities in hiring. For example, we might hire baggage scanners who score higher on measures of conscientiousness or vigilance. We also might be able to train people to better notice rare events (although training benefits do tend to be narrowly tied to the training task and materials; see Simons et al., 2016; Gaspar et al., 2013; McCarley et al., 2004). Under conditions of divided attention or deliberate attentional control, we should expect experts to benefit from their experience (and from any selection effects that enable them to become experts). If individual differences do not predict noticing of truly unexpected objects, however, then we would need to seek other solutions to reduce error rates. We could not rely on such individual differences to select radiologists or nurses who are less likely to miss unexpected objects. Additional training or expertise might help by changing how people search for findings, thereby making what would have been unexpected objects more expected, but training or expertise might not enhance the ability to notice truly unexpected events (Ekelund et al., 2022). If inattentional blindness does contribute to medical errors, we would need to seek alternative solutions such as including additional review by people with different expectations or task goals for whom the critical object would either be task-relevant or potentially expected.

Missed medical findings potentially have many causes. People tend to miss rare, but expected objects (e.g., Mitroff & Biggs, 2014; Wolfe et al., 2007). And, when people are distracted or attempting to multitask, they can miss events that they otherwise would report (e.g., Strayer et al., 2011; Hyman et al., 2010). The published literature includes many examples of missed incidental findings, but many do not specifically address failures of awareness due to inattentional blindness. Some focus on the tendency to miss a second finding once participants have found an initial one, or they address the tendency to notice rare findings. A relatively smaller number of studies have specifically attributed the failure to notice to inattentional blindness. This paper reviews the experimental evidence supporting claims that inattentional blindness contributes to failures of awareness in medicine.

As we will argue, many studies that claim to document evidence of inattentional blindness might not meet all of the criteria necessary to be certain that the task measures inattentional blindness. For example, in one study, participants viewed several radiographs, three of which had an embedded image of a gorilla (Ann-Christin et al., 2018). If participants happened to spot the unexpected gorilla in the first image, then the second and third images would no longer be measures of inattentional blindness because participants would know to look for embedded gorillas while performing their primary task—the gorilla would no longer be unexpected. In inattentional blindness tasks, any trial after participants noticed or were asked to report the presence of something unexpected is considered to measure divided attention, not inattentional blindness.

Similarly, failures of awareness in which participants overlook something they know might be present are not truly demonstrations of inattentional blindness. For example, when participants focused on counting the number of times an instrument was used, many failed to remember how many swabs were left at the surgical site (Pandit et al., 2022). This failure of awareness/memory is not necessarily due to inattentional blindness because participants likely expected swabs to be present.

A retained guidewire is bad for the patient regardless of whether or not it was missed due to inattentional blindness, but if we want to determine whether existing studies provide evidence for inattentional blindness as a contributor to medical error, we need to define it precisely. For a study to be considered a test of inattentional blindness, it must:

- Present an entirely unexpected object. If a study presents multiple “unexpected” objects, it must measure noticing separately for the first occurrence of an unexpected object, and it should make clear that only the first one is unambiguously a test of inattentional blindness.
- Use a primary task that does not require detection of the unexpected object. It should be possible for participants to complete the primary task without noticing the unexpected object. If the unexpected object is directly related to the primary task participants are given, they might devote some attention to the possibility it will be present. For example, if participants are asked to scan a radiograph for a fracture but are told to report anything else anomalous, any other unexpected problem would be related to their performance of the primary task.
- Measure noticing directly by asking participants whether or not they saw the unexpected object or by recording an action that unambiguously reveals noticing.

Many of the studies that claimed to provide evidence for inattentional blindness as a cause of medical error did not fully meet these criteria. Inattentional blindness research in medicine is often constrained by the need to balance experimental control with making the task naturalistic and representative of actual procedures. When documenting a failure to notice something important, the reason for that error is often less critical to the goals of the study (or the patient). Consequently, many of the studies we review fall short of the criteria necessary to draw clear inferences about the contribution of inattentional blindness to medical errors.

In our review, we discuss why studies might not actually have assessed inattentional blindness, and in a separate section, we evaluate whether these studies provide clear evidence for inattentional blindness as a source of medical error. Finally, we provide a set of guidelines for researchers interested in testing whether inattentional blindness contributes to medical errors in a way that is distinct from other types of visual awareness failures.

## Literature search

**Fig. 1:**
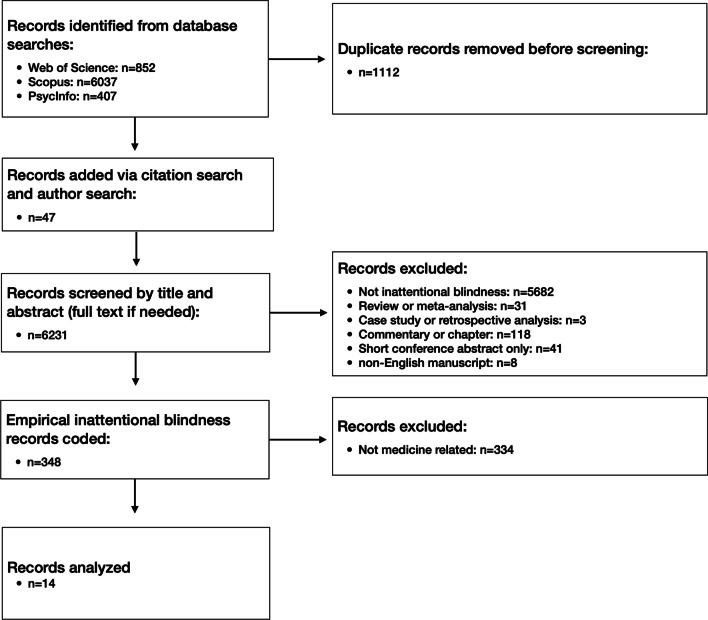
Documentation of our search and exclusion process

As part of a larger literature search for inattentional blindness research (conducted in October 2022; see Simons et al., 2024 for details), we identified all empirical studies that used the terms “inattentional,” “inattentional blindness,” or “attentional blindness” and that were indexed in Web of Science, Scopus, or PsycInfo (see Fig. 1 for documentation of our search and exclusion process). After removing duplicates and supplementing the search with ad hoc reference and author searches, we identified a total of 14 articles reporting empirical research on inattentional blindness in medical domains, including computer-simulated procedures, videos of surgery, reading of radiographs, surgical procedures on model cadavers, and the use of augmented reality during surgery. Each of those 14 articles was reviewed by two of the authors to identify studies reporting inattentional blindness research in a medical context, and each study was then coded by at least two authors. Disagreements in the coding were resolved via discussion with a third coder. The final, complete set of empirical studies of inattentional blindness in a medical context was agreed to by all authors.

For the purpose of inclusion in this review, we used a somewhat liberal criterion for whether or not a study measured inattentional blindness because we wanted to include studies that claimed to measure inattentional blindness but might not have. In our discussion of the included articles, we note where studies might not meet the strict criteria necessary to determine whether a failure of awareness definitively represented a case of inattentional blindness.

Several of these 14 papers also examined differences in noticing as a function of medical expertise. In many ways, medicine is an ideal area to examine expertise effects in inattentional blindness because expertise can be defined based on training and clinical experience, and the materials and tasks used often are directly related to the expertise domain. In non-medicine studies, the evidence for expert/novice differences is mixed (Ekelund et al., 2022). Most of those studies tested small samples, however, and they did not consistently use tasks or unexpected objects relevant to the participant’s expertise domain. One of the larger non-medicine expert/novice studies asked both experienced police officers and police trainees to complete a simulated traffic stop (Simons & Schlosser, 2017). Experienced officers were substantially more likely to notice an unexpected gun on the dashboard in front of the passenger seat.

It is unclear whether greater medical expertise should be expected to increase or decrease the likelihood of noticing unexpected events. One might expect radiologists with greater expertise to be more likely to notice abnormal findings either because the search task itself is easier for them or because they are more familiar with what a normal presentation looks like. If so, anomalies might stand out to them, or they might have additional cognitive resources available to spot the unexpected. Alternatively, novices might be more likely to notice unexpected findings because of their unfamiliarity; novices have a less refined idea of what constitutes a signal and what is noise, so they might devote more attention to “normal” regions of an image or areas unlikely to be problematic, making them more likely to spot anomalies in those areas.

Our review clusters the 14 papers into three groups based on their primary focus. The first covers the effects of using augmentation during surgical procedures on noticing of unexpected issues. The second discusses inattentional blindness in radiology. The third covers inattentional blindness in other medical contexts. In each section, we identify the primary findings in the published literature, and we discuss why some studies might not unambiguously assess inattentional blindness even though they report failures of awareness. We also document reported evidence for expertise effects. Following the review, we provide guidelines and recommendations for study designs that could better document the role of inattentional blindness in medicine.

## Empirical studies

Table 1 provides the citation, type of study, total sample size, and noticing rates for each condition for each of the studies reviewed below.

**Table 1:** Summary of reviewed studies

Study	Study type	Total sample size	Noticing rates by condition:
Dixon et al. (2013)	Augmented reality	32	Overall (noticed something unusual): 12.5% (4/32) Standard endoscopy: 70.6% (12/17) (either object); 41.2% (7/17) (screw); 41.2% (7/17) (complication) Augmented reality: 6.7% (1/15) (either object); 6.7% (1/15) (screw); 0% (0/15) (complication)
Dixon et al. (2014)	Augmented reality	50	Endoscopic with AR Navigation: 28% (7/25) (noticed something unusual); 60% (15/25) (screw); 0% (0/25) (complication) AR as a single display: 16% (4/25) (noticed something unusual); 32% (8/25) (screw); 4% (1/25) (complication)
Hughes-Hallett et al. (2015)	Augmented reality	73	Wireframe overlay: 29% (7/24) (swab); 92% (22/24) (suture) Solid overlay: 20% (5/25) (swab); 88% (22/25) (suture) No overlay: 29% (7/24) (swab); 92% (22/24) (suture)
			High load: 8% (3/39) (swab); 90% (35/39) (suture) Low load: 47% (16/34) (swab); 91% (31/34) (suture)
Marcus et al. (2015)	Augmented reality	50	No image guidance: 90% (9/10) Triplanar display: 40% (4/10) Always-on solid: 20% (2/10) Always-on wire mesh: 40% (4/10) On-demand inverse realism: 40% (4/10)
Ann-Christin et al. (2018)	Radiology	51	(50%) density condition: 9.8% (5/51) (75%) density condition: 19.6% (10/51) (100%) density condition: 19.6% (10/51)
de Cassai et al. (2021)	Radiology	699	4.9% (34/699)
Drew et al. (2013) Study 1	Radiology	24	Radiologists: 16.7% (4/24)
Drew et al. (2013) Study 2	Radiology	25	Novices: 0% (0/25)
Williams et al. (2021) Study 1	Radiology	50	Breast cancer: 34% (17/50) Lymphadenopathy: 70% (35/50)
Williams et al. (2022)	Radiology	74	Noticing rates not reported
Al-Moteri et al. (2018)	Other	40	65% (26/40)
Greig et al. (2014)	Other	142	Overall: 23.9% (34/142) No training: 19.6% (11/56) Advanced: 23.3% (10/43) Expert: 30.2% (13/43)
Ho et al. (2017)	Other	77	Head movement: 66.2% (51/77) Leaky CVC: 32.5% (25/77)
Pandit et al. (2022)	Other	28	Noticing rates not reported
Park and Kim (2021)	Other	47	Noticing rates not reported

For Hughes-Hallett et al. (2015), subjects in each overlay group were assigned to one of two additional conditions (high or low load)

### Surgery and augmented reality

Augmented reality (AR) is increasingly used by surgeons in the operating room (Vávra et al., 2017). By superimposing a computer-enhanced image on the body, surgeons can merge the patient’s anatomy with medical imagery such as CT scans. Much as a GPS overlay helps drivers or pilots navigate their routes (Alexander et al., 2005), augmented reality allows surgeons to operate with greater precision. Based on research in domains other than medicine, though, the use of augmented reality has consequences for visual awareness, including an increased risk of missing unexpected events. For example, drivers using an augmented reality head-up display are less likely to see pedestrians crossing the road (Wang et al., 2022). Pilots using head-mounted augmented displays better adhered to the target flight path but were more likely to miss critical, unexpected events (Wickens & Alexander, 2009). This pattern might apply in medicine as well.

Four of the 14 papers identified in our search examined whether the use of augmented reality as a surgical aid increases rates of inattentional blindness. In these studies, participants watched videos or performed simulated surgical procedures in which augmented reality was used and unexpected stimuli were present. One study also investigated whether cognitive load contributed to increased inattentional blindness when using augmented reality.

In one study, otolaryngology surgeons or trainees attempted to maneuver an endoscopic camera to a target location in a model cadaver (Dixon et al., 2013). They either used a standard camera display or an augmented view showing anatomic contours (i.e., the outline of internal structures such as the carotid arteries and optic nerves). The cadaver included two unexpected elements: a foreign body (a screw) and a critical complication (the optic nerve draped into the sinus). Neither was mentioned to the participants and neither was directly relevant to the navigation task. After completing the navigation task, participants were asked four questions of increasing specificity to determine whether they had noticed the unexpected stimuli (from “Did you notice anything unusual?” to “Did you notice a screw?”). Overall, a majority of participants missed both the screw and the complication, and only 4 of them reported noticing something unusual. However, noticing rates were higher when researchers asked about specific findings. The screw and complication were noticed at comparable rates under traditional viewing (screw: 7/17 or 41.2%; complication: 7/17 or 41.2%). Those who used an augmented display performed the navigation task more accurately, but they also were far more likely to miss the unexpected screw and optic nerve complication (screw: 1/15 or 6.7%; complication: 0/15 or 0%). Overall, only 6.7% (1/15) noticed at least one object in the AR condition compared to 70.6% (12/17) with a traditional display. This result mirrors findings with pilots that augmented reality can improve navigation while impairing the detection of unexpected objects and events (Wickens & Alexander, 2009). The study found no difference in noticing rates when comparing surgeons and fellows, residents in their first and second year, or residents in their third through fifth years, although the number of participants in each group was too small to draw definitive conclusions.

The use of two unexpected objects raises challenges for interpreting the results as evidence of inattentional blindness, however. If a participant noticed the first object, the second one would no longer be entirely unexpected. Given that the two unexpected objects were present simultaneously, there is no way to determine which was noticed first for those participants who happened to notice both. The study also did not specify which of the four questions was used as the primary measure to determine the noticing rate (and results varied based on the question), although it did provide noticing rates separately for each question.

In another augmented reality “navigation” study using the same unexpected stimuli, otolaryngologists and residents with significant endoscopic experience used either a single, augmented head-up display or a standard endoscopic display coupled with navigation information presented on a second monitor rather than superimposed on the body (Dixon et al., 2014). Noticing was assessed the same way as in the earlier study (Dixon et al., 2013), but again it was unclear which question was used to count someone as having noticed. Here, the screw was noticed more than the complication, possibly because the screw was more distinct from the background whereas the optic nerve issue was less salient compared to the previous study (screw: 8/25 or 32% (AR) and 15/25 or 60% (submonitor); complication: 1/25 or 4% (AR) and 0/25 or 0% (submonitor)). Participants who used the augmented single display were less likely to report noticing anything unusual (4/25, or 16%) than were those using two separate displays (7/25 or 28%). Noticing was comparable for surgeons and trainees, but the sample sizes again were too small to reliably measure expertise effects. Unlike the 2013 study, navigation performance was no better with augmented reality, perhaps because the same information was available to both groups.

Another augmented reality endoscopic navigation study examined whether different types of visual overlay had differing impacts on noticing (Marcus et al., 2015). Medical students and junior doctors with no endoscopic experience used a probe in a model head and attempted to identify a basilar tip aneurysm (i.e., a bulge in the basilar artery in the brain). The unexpected object was a surgical clip.

Some participants had no image guidance, some had a conventional triplanar display (three views of the brain displayed on a separate screen), and some used one of three different types of augmented reality display: always-on solid; always-on wire mesh; or on-demand inverse realism (anatomic contours are present but partially transparent). All forms of image guidance improved precision and reduced task time, but they also resulted in more people missing the surgical clip. Fewer than half of the participants in the triplanar display or any of the augmentation groups noticed the clip (triplanar: 4/10; solid: 2/10; wire mesh: 4/10; inverse realism: 4/10), whereas 9 out of 10 participants in the no-augmentation condition noticed it. Given the small number of participants in each condition, we cannot draw strong conclusions about the effects of different forms of augmentation on noticing, but the pattern of lower noticing rates with augmentation than without it is consistent with the other studies in our review. Note that the paper did not explain how noticing was determined—it stated only that participants were prompted.

A final study of augmented image guidance tested surgeons and surgical residents (averaging eight years of postgraduate experience) while they watched a video of surgery (Hughes-Hallet et al., 2015). The unexpected objects were two foreign bodies visible within the operative scene: a swab in the periphery and a suture in the center. Participants were assigned to one of the six combinations of cognitive load (high vs. low) and augmentation (none, wireframe, solid). In the high-load conditions, participants counted the movements of two surgical instruments, while in the low-load conditions, they watched the video without performing a specific primary task. Unlike the other augmentation studies, this study found no substantial differences in noticing of unexpected objects with and without augmentation. Across the three overlay groups (combining the cognitive load conditions), approximately 25% noticed the peripheral swab and approximately 90% noticed the central suture (no augmentation: 7/24 and 22/24; wireframe: 7/24 and 22/24; solid: 5/25 and 22/25).

Why did augmented reality have no effect on noticing in this study? The authors argue that in previous studies, participants using augmented reality had increased cognitive load. For example, participants in the Dixon et al., (2013, 2014) studies needed to “maintain the optically tracked probe and reference arc in line of sight of the camera” when using image guidance (Hughes-Hallet et al., 2015). Increasing task difficulty (cognitive load) while keeping the visual display constant increases inattentional blindness (Chabris et al., 2011; Fougnie & Marois, 2007; Simons & Chabris, 1999). Cognitive load was reduced in this study because participants did not need to manage the equipment themselves. Consistent with this idea, when combining across the overlay conditions, noticing rates were lower for people in the high-load conditions (swab: 3/39 or 8%; suture: 35/39 or 90%) than in the low-load conditions (swab: 16/34 or 47%; suture: 31/34 or 91%), at least for the swab. Future research should seek to systematically disentangle the effects of cognitive load and augmented reality on inattentional blindness in surgery.

Although this study examined a possible explanation for the costs of augmented reality, as in the studies by Dixon et al., (2013, 2014), only the first detected object provides a measure of inattentional blindness. Again, though, the paper did not separately report the detection of the first unexpected object noticed. So, as for the first two augmented reality studies, the actual noticing rates under conditions of inattentional blindness might be different if we could analyze only the first unexpected object that was detected.

### Radiology and visual search

Visual search is central to the tasks that radiologists perform daily, and many of the principles revealed from basic research using simplified laboratory displays appear to generalize to search in radiological displays (Wolfe, 1995). In traditional laboratory tasks, participants search through an array of objects, letters, or shapes in an attempt to find one or more target items amidst the many distractors or irrelevant items. Similarly, radiologists must locate target problems against the background “noise” of the rest of the image. Unlike laboratory search tasks, though, failures of awareness in radiology can have serious consequences. Studies of attention in radiology search have revealed a number of factors that influence awareness, not all of which necessarily reflect inattentional blindness. For example, noticing one abnormality in an image can make it less likely that radiologists will notice additional findings (e.g., Berbaum et al., 2001).

Our search identified 5 papers that specifically investigated inattentional blindness in radiology. In these studies, participants searched for medical abnormalities in images that also contained unexpected objects, some of which were clinically relevant, incidental findings (e.g., a large breast mass), and others of which were not (e.g., a cartoon gorilla).

In perhaps the best-known study of inattentional blindness in radiology (Drew et al., 2013), participants scanned through five sets of CT images on a computer while searching for lung nodules (i.e., an abnormal growth). The final set of CT images embedded an image of a gorilla that was 48 times the size of an average nodule. Not surprisingly, radiologists outperformed participants who lacked medical training in detecting lung nodules, but both groups missed the gorilla (radiologists in study 1: 20/24 or 83.3% missed the gorilla; novices in study 2: 25/25 or 100% missed the gorilla). Eye tracking data showed that more than half of the radiologists who missed the gorilla looked directly at it. Given how few of the participants in either group noticed the gorilla (only 4/49 or 8.2%), the study does not provide clear evidence about whether expertise affected noticing. (Note that the researchers asked participants three questions of increasing specificity about what they noticed during the critical trial, but the exact criteria used to code someone as having noticed or not were unspecified.)

Drew and colleagues might have been the first to embed a gorilla into medical images, but others followed their example. Groups of 2nd and 4th-year chiropractic students who were familiar with plain film radiographs viewed a series of 20 radiographs projected onto a screen (Ann-Christen et al., 2018). One of the 20 images showed a gorilla at 50% tissue density, another at 75% density, and a third at 100% density (when density is higher, the tissue appears lighter and the darker gorilla image stands out more).

Noticing rates were lowest for the 50% density gorilla (5/51 or 9.8%) and slightly higher for the 75% (10/51 or 19.6%) and 100% (10/51 or 19.6%) gorillas. Fourth-year students were more likely than 2nd-year students to notice the 75% (9/26 or 24.6% vs. 1/25 or 4%) and 100% density gorillas (9/26 or 24.6% vs. 1/25 or 4%) but not the 50% density gorilla (3/26 or 11.5% vs. 2/25 or 8%). Consistent with Drew et al. (2013), noticing rates were slightly higher for the more experienced participants, but again the sample sizes were small enough that any claims of experience effects should be treated as tentative.

Interpreting the results from this study as a demonstration of inattentional blindness is challenging for several reasons. First, all participants experienced all three gorilla images, so those who spotted the gorilla after one or two images might have looked for it subsequently. Given that the participants all completed the study simultaneously and that the images were shown on a projection screen, whichever gorilla image was shown first was the critical trial. However, the authors did not report noticing rates separately for the image presented first. From the reported numbers, we can infer that inattentional blindness for the first gorilla was either 90.2% or 80.4%, depending on whether the first image shown was the 50% density one or either the 75% or 100% density one (which had the same overall noticing rate).

Second, prior to performing the task, participants completed a questionnaire that asked them to report any findings after each image was presented (the specific criteria used to assess noticing from these responses was not presented). The final question on the survey asked if they were familiar with the study, “The Invisible Gorilla Strikes Again” (Drew et al., 2013). If participants happened to peruse the survey before starting the task, they would have had reason to anticipate a gorilla in the images. However, the paper reported no difference in noticing when people did or did not report familiarity with the Drew et al. (2013) study.

In a final gorilla-in-a-radiograph study, participants completed an online survey about five simulated cases of the management of surgeries by anesthesiologists and residents (de Cassai et al., 2021). Each case described a simulated patient’s medical history, medications, proposed surgery, electrocardiogram, chest radiography, and preoperative blood testing. Participants answered questions after each case presentation. In the fifth case, the head of a gorilla was embedded inside the shadow cast by the heart and surrounding membrane in the chest radiograph. At the end of the entire survey, participants were asked if they had any comments on the final case, and they were counted as noticing the gorilla if they reported seeing an animal or an abnormality in the cardiac shadow. (The paper mentioned that there were additional criteria used to determine noticing but did not specify what those were.) Consistent with the low noticing rates in other studies, only 4.9% of participants (34/699) reported the gorilla.

Unlike earlier studies, the total sample size in this study was large enough to measure the effects of experience, but with only 34 people reporting the gorilla and the remaining 665 not reporting it, the estimated noticing percentages are inherently noisy. In general, there was little difference between different groups of practitioners or between experienced and inexperienced respondents, regardless of how such differences were measured: anesthesiologists (23/547 or 4.2%), residents (11/152 or 7.2%), practitioners at private hospitals (2/116 or 1.7%), public teaching hospitals (21/325 or 6.5%), practitioners at public non-teaching hospitals (11/258 or 4.3%), those with 0–5 years of experience (9/168 or 5.4%), those with 6–10 years of experience (6/111 or 5.4%), those with 11–15 years of experience (6/194 or 3.1%), and those with 16 or more years of experience (2/74 or 2.7%).

The two other radiology studies of inattentional blindness examined failures to notice clinically relevant issues rather than gorillas. In one study, radiologists screened for and clicked on lung nodules in seven chest CT scans (Williams et al., 2021). Three of the seven CT images contained lung nodules, and the final scan contained an unexpected large breast mass and swollen lymph nodes. Many radiologists failed to notice these incidental problems. Overall, 66% of the radiologists (33/50) failed to report the breast mass and 30% (15/50) failed to notice signs of swollen lymph nodes. The breast mass may have been noticed less often because it appeared outside of the chest wall, while the subjects were looking for lung nodules within the chest wall. These noticing rates were higher than in the other studies, perhaps because these additional findings were clinically relevant. Noticing rates reportedly were not predicted by years of experience or the number of chest CTs read per week. Again, because the study contained two incidental findings, we cannot determine the noticing rate for just the first one, and noticing one problem might inspire people to look for others (although satisfaction of search might also lead people to stop looking after finding one such object; see Berbaum et al., 2001). (We did not include study 2 from this paper because it was not designed as a test of inattentional blindness: Participants were given a list of possible stimuli they might encounter, so participants knew to look for a possible breast mass or swollen lymph nodes.)

The final radiology study asked radiology residents, fellows, and attending physicians to view abdominal CT scans (Williams et al., 2022). Participants were told either that the patients were liver donors (*n* = 18) or kidney donors (n = 19), so one organ was the likely focus of their attention and the other was not. With this design, a failure to notice anomalies in the uncued organ could be considered inattentional blindness, but anomalies in the cued organ would not be (because it was the focus of their attention). We cannot be certain, however; monitoring for incidental findings is a key aspect of radiological practice, so participants might devote some attention to looking for abnormalities outside of the cued organ, making the task one of divided attention. Studies in which the unexpected object falls outside of the typical incidental findings a radiologist might expect (e.g., Drew et al., 2013) might better measure inattentional blindness even if they are less representative of the sorts of incidental findings most relevant to radiological practice.

Participants in this study each viewed four normal cases, two with kidney abnormalities, and two with liver abnormalities. For each case, they were asked to report any concerning regions and to explain why they were of concern. The paper did not describe the criteria used to measure noticing, and it did not report the proportion of participants who noticed each abnormality. Additionally, although the paper acknowledges that increasing the number of unexpected events can cause participants to “expect the unexpected,” it did not report the order in which the cases were presented or the noticing rate for the first anomaly encountered. Instead, it reported the total number of abnormalities detected. The authors also note that even the first critical event may have been rendered somewhat expected by the presence and detection of additional anomalies outside of the organs of interest in the non-critical trials. Participants reportedly detected an average of 44% (1.76/4) of abnormalities, with no significant difference in the number of abnormalities detected in the cued and uncued organ (despite more thorough searching of the cued organ). The paper also reported no differences as a function of expertise.

### Other medical studies

A final set of 5 studies examined inattentional blindness in medical contexts other than radiology and without using augmented reality navigation aids. Two used patient-monitoring simulations and three asked participants to watch videos of a patient.

Both patient-monitoring studies assessed nursing students who were presented with signs that a patient was experiencing a problem. The first study examined whether nursing students would detect the unexpected occurrence of progressive hypovolemic shock (i.e., the severe loss of blood and bodily fluid), identifiable from changes in the patient’s vital signs during a simulated training program (Al-Moteri et al., 2018). The authors measured noticing by assessing whether or not the students looked at the relevant vital signs, but fixation alone is a poor proxy for noticing because participants can look right at an unexpected object and not report it (Beanland & Pammer, 2010; Drew et al., 2013; Memmert, 2006)—looking is not the same as seeing. Although they coded fixation as their measure of inattentional blindness, the authors acknowledge that “Inattentional blindness is mainly evidenced by a failure of the individual to respond to the deterioration cues.” Rather than using the looking measure as an indication of inattentional blindness, we instead used what the paper referred to as a “recognition failure”—a failure to address the problem despite having fixated on the relevant part of the simulation for 200 ms or longer. Nearly 2/3 of participants (26/40 or 65%) exhibited inattentional blindness based on this criterion. Although the authors reported no relationship between training experience and inattentional blindness for their fixation measure, they did not report tests of the relationship between training and their recognition failure measure.

In the second patient-monitoring study (Park & Kim, 2021), nursing students monitored a mannequin simulator of a patient with chronic obstructive pulmonary disease (COPD) for 20 min for worsening symptoms and were asked to respond appropriately. The 47 participants in the experimental condition were also shown other problems that were irrelevant to the patient’s underlying disease, including the presence of a tube in the patient that was not provided by the ER nurse, an incorrectly set infusion speed in a pump, improperly functioning wall oxygen, an infusion status that suggested a medication error, and an error in the number of remaining drugs provided. The primary purpose of the study was to examine whether a simulation including these other, unrelated problems might be a useful educational tool for nursing students, increasing their situational awareness. The authors found that the incorporation of these additional events in the simulation improved aspects of situational awareness. Unfortunately, given the focus on overall situation awareness and patient care, the paper did not report whether or not patients noticed any of these unrelated events. Instead, the paper only reported differences in a situation awareness measure between nurses who experienced these additional events and those who did not. From that overall measure of situational awareness, we cannot infer whether or not people noticed any given event (although the paper does note that some students were confident that there had not been an unexpected tube placed in the patient until they were shown a video of it during the debriefing). Moreover, the presence of multiple issues means that spotting one problem might lead nurses to look for others, in which case any subsequent noticing or missing would not assess inattentional blindness.

In the first video-viewing study (Pandit et al., 2022), neurosurgeons watched two videos of a surgical procedure. The first showed a spinal surgery that was familiar to the surgeons and the second showed a less-familiar procedure. The participants were either asked to focus on part of the procedure by counting the number of times an instrument was used or to simply watch the video without performing an additional task. They then were asked for a count of the number of swabs left in the surgical site, something that they had not been informed about in advance. The authors note that “After the first video was completed, both groups were now aware of the experiment’s motive and likely to be more vigilant for other unexpected distractors.” Hence, as measures of inattentional blindness require entirely unexpected objects, the second video would not constitute an inattentional blindness trial because participants knew they might be asked about the swabs or other aspects of the scene not relevant to their primary task. The study design compared surgeons who had undergone a mindfulness training program (*n* = 13) to surgeons who had not (*n* = 15). The paper reported that the groups differed in the number of swabs reported, with greater error for the control group than the mindfulness group. Unfortunately, incomplete reporting makes it difficult to determine the proportion of people who noticed. The paper provides the error rate for the reported count of swabs, but it does not report the actual number of swabs in the video (the authors provide a link to the video, but do not specify which 90-s segment participants viewed, so this information cannot be determined), nor does it report the exact questions asked of participants. Asking how many swabs were left in the patient likely would result in some participants guessing a number, so it does not make clear whether any of the swabs had been detected. It also was unclear from the description whether the swabs were truly unexpected in the context of this surgical procedure. That is, would surgeons who are familiar with a procedure know that some swabs would be present during it? For those reasons, it is unclear whether this study truly measured inattentional blindness, and if it did, what the rate of noticing was.

The other two video-viewing studies involved monitoring of a patient’s status. In the first, upper-level medical students and certified anesthesiologists watched a video of a simulated septic patient having their small and large intestines removed (Ho et al., 2017). They were asked to report any abnormalities they noticed, and several occurred that were directly related to their presumed role as anesthesiologists. In addition to those abnormalities, two other unusual events occurred: patient head movement and a leaky central line catheter. Similar to radiologists looking for incidental findings, the participants in this study were likely looking for cardiorespiratory issues and not these sorts of anomalies (they were given a list of normal cardiorespiratory parameters), so we treated failures to notice the head movement and leaky catheter as unexpected even though they technically were reportable events according to the instructions given to participants. However, the authors do note that such events “may be unexpected to anesthesiologists but not necessarily to the same degree to students” who may be less selective when discriminating signal from noise due to their relative lack of experience.

Overall, 83% of the medical students (38/46) noticed the head movement, but only 42% of the anesthesiologists (13/31) did. For the leaky catheter, the difference in noticing between medical students (18/46 or 39.1%) and anesthesiologists (7/31 or 22.6%) was smaller. Unfortunately, the paper did not report noticing rates for the first unexpected event separately, and it is not possible from what was reported to extract that information (in part because the two unexpected events occurred simultaneously).

The last study in this set investigated the effect of resuscitation experience on noticing that a patient’s oxygen supply had been disconnected (Greig et al., 2014). Participants watched a short video depicting a simulated cardiac arrest. The video included a number of other unexpected events, including changes to the clothing and equipment of the team assisting the patient (meant to test for change blindness). After watching the video, participants were asked if they noticed anything unexpected or unusual and answered using an open-ended, free-text response. They were then asked to tick boxes next to any events that they noticed in a list (e.g., “The cardiac rhythm changed” and “The oxygen malfunctioned”). The latter measure was used for the primary analysis. Overall, 76.1% (108/142) of participants missed the disconnected oxygen supply, and those with more training were slightly more likely to notice: 20% of those with basic or no training (11/56); 23% of those with advanced training (10/43), and 30% of experts (13/43). But even the most clinically significant events were frequently missed by experts. Given that this video showed multiple unexpected events prior to the primary ones of interest, if participants detected any of those changes the study would not provide a test of inattentional blindness because that participant would know to search for other “unexpected” events.

## Guidelines for future work

This set of 14 papers provides clear evidence that surgeons, radiologists, and nurses can miss critical, medically relevant objects and diagnoses when engaged in attention-demanding tasks like surgery, monitoring patients, or reading radiographs. The role of expertise remains uncertain, primarily due to the small number of participants in most studies. The results of using augmentation mirror earlier findings with pilots: Surgeons can navigate more efficiently with augmentation, but they are less likely to notice other problems. Collectively, these studies suggest that a failure to notice task-irrelevant objects can be a key source of medical error, but it is less clear whether those failures represent inattentional blindness or a failure of divided attention.

In our introduction, we established a set of criteria necessary for a failure of awareness to constitute evidence of inattentional blindness. And, in our review of each article, we noted some ways in which these studies did not distinguish inattentional blindness from other mechanisms. Table 2 lists all of the reviewed articles and indicates why each might not have truly measured inattentional blindness. All of these studies either explicitly aimed to study inattentional blindness or strongly implied that doing so was a goal. Some might well have tested inattentional blindness, but they did not provide sufficient information to verify how awareness was measured or whether the critical object was truly unexpected. Nonetheless, even if a study measured errors of divided attention rather than inattentional blindness, it still could have important implications for patient care. Incidental findings missed because of divided attention might well be the more common type of awareness failure in medicine. Each of the reviewed studies contributes to our understanding of awareness failures in medicine despite the uncertainty about the underlying mechanisms. In the remainder of this section, we discuss the challenges in verifying that a study truly does measure inattentional blindness, and we provide recommendations for researchers interested in determining the contribution of inattentional blindness to medical errors.

**Table 2:** Reasons why it is challenging to determine whether failures to notice resulted from inattentional blindness

Study	Not clear how many participants noticed	Measured noticing only indirectly	Unclear what criterion was used to assess noticing	Possible that the critical object was expected	Primary task required reporting of “unexpected” events	Included multiple unexpected events without separately reporting noticing rates for the first one
Al-Moteri et al. (2018)		X		X	X	
Ann-Christin et al. (2018)			X	X	X	X
de Cassai et al. (2021)			X		X	
Dixon et al. (2013)			X	X		X
Dixon et al. (2014)			X	X		X
Drew et al. (2013) Study 1			X			
Drew et al. (2013) Study 2			X			
Greig et al. (2014)				X	X	X
Ho et al. (2017)				X	X	X
Hughes-Hallett et al. (2015)			X	X		X
Marcus et al. (2015)			X			
Pandit et al. (2022)	X			X		X
Park and Kim (2021)	X	X	X	X	X	X
Williams et al. (2021) Study 1				X		X
Williams et al. (2022)	X		X	X	X	X

### Considerations for the unexpected object or event

To ensure that a study measures inattentional blindness, experiments should avoid alerting participants that something unusual will happen. If participants allocate some amount of attention to monitoring for something “unexpected,” they are no longer being studied under conditions of inattention. Studies also should not mention the specific unexpected stimulus at any point before or during the trial. For example, one study asked participants if they were familiar with “The Invisible Gorilla Strikes Again” study (Drew et al., 2013) at the end of a survey that participants had access to during the trials (Ann-Christin et al., 2018). If participants saw this survey item, they might have expected a gorilla, making the study a test of divided attention, not inattentional blindness.

Even when the study does not explicitly mention the unexpected object, the methods might well induce participants to “expect the unexpected,” which again makes the task one of divided attention rather than inattention. For example, one study included several trials of a change detection task prior to the inattentional blindness trial (e.g., Greig et al., 2014). If participants know that unexpected events might occur (e.g., changes), they won’t focus exclusively on the primary task. Instead, they will devote some attention to detecting changes or other unexpected elements.

The most common issue with interpreting the documented failures of awareness as inattentional blindness was the use of multiple unexpected objects or multiple trials with purportedly unexpected objects. All studies of inattentional blindness in augmented reality but one (Dixon et al., 2013, 2014; Hughes-Hallett et al., 2015) and several in other domains (Ann-Christin et al., 2018; Greig et al., 2014; Ho et al., 2017; Pandit et al., 2022; Park & Kim, 2021; Williams et al., 2021, 2022) used multiple unexpected objects. If people detect an unexpected object, they then will know that such objects are a possibility and might search for other out-of-place objects—once participants are asked if they noticed a gorilla, they’ll look for gorillas the next time they are asked to count passes of a basketball (Simons, 2010). That shift in strategy means that other objects will be detected under conditions of divided attention rather than inattention. Using a single critical trial with one unexpected object allows for a clearer inference that the object was unattended. A few studies acknowledged that the use of multiple trials with “unexpected” events could render subsequent events somewhat expected (e.g., Pandit et al., 2022; Williams et al., 2022). If so, then these subsequent events measure divided attention instead of inattentional blindness.

Using multiple critical objects also muddies the interpretation of noticing rates for two reasons. First, if people spot one unexpected object and begin searching for others, that would likely inflate noticing rates for the second object due to the contribution of expectations and attention (rather than inattention). Second, with two objects present, measures of noticing for the *first* detected object can be inflated relative to a case in which there is only one object present. To illustrate, imagine a study in which either a screw or a guidewire was unexpectedly present in a cadaver as participants practiced a surgical procedure. Let us assume that if only one of those objects were present, it would be noticed by 50% of participants. If both a screw and a guidewire were present simultaneously, participants potentially could spot either object first, increasing their chances of noticing *something* unusual relative to the case in which only one object was present. Consequently, with multiple objects, we would expect the noticing rate for the first detected object (either the screw or the guidewire) to be higher than if only one object were present. Before participants noticed either object, they would still be operating under conditions conducive to inattentional blindness because whichever object they happened to notice first was unexpected and not part of their search goals. But comparing noticing rates for that object to noticing with only one unexpected object present is problematic unless we know the separate likelihoods of noticing each object on its own.

A study using multiple critical objects to measure rates of inattentional blindness must, at a minimum, report the noticing rate for the first object detected. Ideally, it should also track the proportion of times that each object was detected first and acknowledge that noticing rates might be inflated compared to a situation in which only one unexpected object was present. Combining noticing across both objects (or reporting noticing rates for each object individually without noting which was detected first) does not provide a measure of noticing under conditions of inattentional blindness because the noticing rates for both the first and second object likely will be inflated for different reasons (two chances to detect the first one and deliberate search for other objects).

Another common issue involves the need to eliminate any expectation that a critical object might be present. In some studies, the critical objects were directly related to the primary task participants were performing. Consequently, participants likely were devoting some attention to their possible presence, so it is not truly a test of inattentional blindness. For example, one study asked anesthesiologists to “highlight remarkable findings” when looking at simulated cases of surgical patients, meaning that any abnormality would be expected to some degree (de Cassai et al., 2021). Given that monitoring for incidental findings is generally expected of medical practitioners, these sorts of awareness failures are ecologically relevant and important to understand. However, they might not reflect inattentional blindness.

To provide an unambiguous test of inattentional blindness, the unexpected object should not be directly related to the primary task given to the participants. For radiologists, the task could be to search for a particular type of lesion and the unexpected object could be an entirely different type of problem, such as looking for lung nodules and missing an unexpected gorilla image (Drew et al., 2013). If radiologists were not looking for gorillas and were not asked to report *any* abnormality they saw, they had no reason to look for the gorilla.

A related issue arises when the critical object is irrelevant to the primary task given to participants, but still typical for that medical context. For example, in one of the reviewed studies (see Pandit et al., 2022), surgeons viewing a video of a procedure might expect surgical swabs to be present even if their task was to count how often the surgeon used a tool, so they might have deliberately devoted some attention to the swabs while viewing the procedure. In another case, even if participants are instructed to look specifically for lung nodules in a CT scan, incidental findings such as a breast mass or swollen lymph nodes might be somewhat expected if they occasionally appear in such scans (Williams et al., 2021).

Of course, the use of something entirely unexpected, like a gorilla, makes that particular failure of awareness less relevant to the sorts of errors that actually occur in practice, even if it might provide a better measure of inattentional blindness. Researchers need to balance their desire to understand realistic and typical medical errors (missed lesions, missed swabs) against the goal of evaluating whether such failures reflect inattentional blindness as opposed to a different mechanism. There is nothing inherently problematic about examining naturalistic errors, but they can make it more difficult to determine the underlying mechanism.

### Criteria for noticing and reporting of noticing rates

To determine the noticing rate under conditions of inattentional blindness, noticing or missing of an object must be measured directly (e.g., via an explicit report or action) and not assumed or inferred. The need for precise reporting applies more broadly, though, regardless of whether noticing failures are due to inattentional blindness or to some other failure. Several papers in our review did not use a direct measure of noticing. For example, Park and Kim (2021) tested nursing students in patient-deterioration simulation trials, but they did not report noticing rates for unexpected events (e.g., indications of an error in medication administration) and instead reported whether the presence of such events affected a measure of situational awareness (note, though, that their primary goal was to determine whether simulations using events for which inattentional blindness might occur could improve situational awareness, so determining noticing rates may not have been necessary for their purpose). Another study used fixation on abnormal vital signs (indicating hypovolemic shock) as an indication that participants noticed them (Al-Moteri et al., 2018). Yet fixation alone does not mean that people noticed (Beanland & Pammer, 2010; Drew et al., 2013; Memmert, 2006). Another study (Pandit et al., 2022) asked surgeons to report how many swabs were left in a surgical site and reported the median error across groups of surgeons in different conditions, but it did not report the number of participants who noticed a swab (perhaps because the swabs were expected, in which case the study would not be measuring inattentional blindness). It is imperative that noticing be measured as directly as possible when assessing inattentional blindness.

In addition to reporting noticing rates, studies also need to specify the criteria used to determine whether or not someone noticed. Almost all of the reviewed studies gave incomplete descriptions of what constituted noticing. Dixon et al., (2013, 2014), for example, asked a set of four questions with varying specificity and provided data for the responses to each (a good approach), but did not specify which was used as a primary measure to code participants as having noticed or missed the unexpected object. When noticing is measured in multiple ways, it is important to specify the primary criterion used to label someone as noticing or missing (and ideally to report whether the pattern of results is robust across different criteria). In an example of more complete, precise reporting, one study noted that “observers were presented with a list of potential events and asked to select any that they witnessed” and provided a table showing the prompt and list of events (Greig et al., 2014).

For inattentional blindness studies, it is straightforward to report all of the relevant data in the paper itself. Noticing is a binary measure (noticed or missed), so reporting the percentage of noticers along with the ratio of the noticers to the total number of participants in each condition (e.g., “34 of 142 participants (23.9%) noticed”) provides all of the relevant data, making it possible for future analysts to meta-analyze noticing rates or to combine data from multiple studies. Studies of inattentional blindness or any other failure of awareness should provide noticing rates and numbers of participants noticing and missing separately for each between-participants condition (e.g., experts and novices) so that all of the data are fully conveyed in the paper itself. If the study includes multiple critical objects, the noticing rate should be presented separately for the first unexpected object encountered (in addition to reporting noticing rates for each of the objects).

## Constraints and limitations

Given that our literature search was limited to papers that used the terms “inattentional,” “inattentional blindness,” or “attentional blindness,” it is possible that studies of inattentional blindness that did not use any of these terms might have been missed, especially considering that such studies likely would have been published by medical researchers who might be unfamiliar with the term. We did examine the citations of the included papers to search for additional studies, but we found no more relevant empirical papers. At a reviewer’s suggestion, we also searched on Scopus using the search terms [incidental & error & radiology & (experiment | empirical)] and identified approximately 100 records, but those did not include any inattentional blindness experiments that we had not already found.

Many of the included papers had designs, analyses, or reporting that made it unclear whether they measured inattentional blindness or a different sort of awareness failure. With more complete reporting, some potentially could have provided more compelling evidence that they measured inattentional blindness. Others might have measured inattentional blindness but the use of multiple objects made it difficult to evaluate the noticing rates.

## Conclusion

Failure to identify clinically relevant but unexpected events can have important consequences for patients. Our review suggests that inattentional blindness is a relevant failure of visual awareness in medical error. Although augmented reality tools may enhance surgical precision and speed, they might impair the surgeon’s ability to notice unexpected events. In radiology, inattentional blindness appears to contribute to missing incidental findings. The effect of expertise on noticing is less clear. A few studies reported greater noticing by novices, others reported more noticing by experts, and some reported no relationship between noticing and experience. Many of the reviewed studies may have assessed failures of awareness under conditions of divided attention rather than actually measuring inattentional blindness, so further studies that directly test the contributions of inattentional blindness to medical error are needed. We hope the guidelines we have provided will lead to studies that more directly assess the contribution of inattentional blindness to medical errors.

## Data Availability

Data sharing is not applicable to this article. No datasets were generated or analyzed as part of this review.
